# Imaging comparisons of cystic echinococcosis in HIV-positive and HIV-negative patients

**DOI:** 10.4102/sajr.v30i1.3493

**Published:** 2026-08-24

**Authors:** Kate Couzens-Bohlin, Gercois Human, Elsabe Smit, Urda Kotze, Jake Krige, Christo Kloppers, Marc Bernon, Stefano Cacciatore, Eduard Jonas

**Affiliations:** 1 Surgical Gastroenterology Unit, Department of Surgery, Faculty of Health Sciences, University of Cape Town, Cape Town, South Africa; 2 Groote Schuur Hospital, Cape Town, South Africa; 3 Department of Radiology, Faculty of Health Sciences, University of Cape Town, Cape Town, South Africa; 4 Bioinformatics Unit, International Centre for Genetic Engineering and Biotechnology (ICGEB), Cape Town, South Africa

**Keywords:** cystic echinococcosis, *Echinococcus granulosus*, hydatid, HIV, radiology, imaging

## Abstract

**Background:**

Radiological imaging is crucial for diagnosing hepatic cystic echinococcosis (HCE) and assessing complications. Case studies suggest HCE with human immunodeficiency virus (HIV) co-infection is associated with larger, more multilocular cysts, increased extrahepatic dissemination, rupture, secondary infection and biliary invasion.

**Objectives:**

To compare the radiological presentation of HCE in HIV-positive (HIV+) and HIV-negative (HIV-) patients and determine whether HIV co-infection influences imaging features.

**Method:**

Pre-operative imaging (chest X-rays, ultrasound, CT and MRI of HIV+ and HIV-HCE patients at our tertiary facility between 2011 and 2023 was reviewed. Cyst characteristics and disease staging (WHO and Gharbi classifications) were independently assessed by two blinded radiology specialists.

**Results:**

Eighty-six patients (45 HIV+, 63 females) were included. Single unilocular cysts predominated (37.8% HIV+ and 36.6% HIV-). Extrahepatic cysts occurred in 40% of HIV+ and 34.1% of HIV- patients. Right hepatic lobe involvement was more common in HIV+ patients (86% vs. 62.5%). Median cyst diameter was 14.2cm in HIV+ vs. 11.3cm in HIV- patients. Rates of biliary communication, bile duct dilatation, intraperitoneal cyst rupture and cyst infection were similar in HIV+ versus HIV- patients (11.1% vs 12.2%, 11.1% vs 17.1%, 20% vs. 26.8% and 8.9% vs. 19.5%, respectively), as was WHO and Gharbi stage distributions.

**Conclusion:**

HIV prevalence (52.3%) exceeded the national prevalence (12.7%), supporting hypotheses that HCE may self-limit less frequently in HIV+ patients. However, co-infection was not associated with more advanced or complicated radiological disease.

**Contribution:**

Imaging remains central to HCE diagnosis and management, particularly in HIV+ individuals, where serological testing is less reliable.

## Introduction

The cestode *Echinococcus granulosus* (EG) causes cystic echinococcosis (CE), defined by the World Health Organization (WHO) as a tropical neglected disease.^1^ Humans are an accidental intermediary final host for EG, which normally follows a sheep-dog host cycle.^2,3^ Cystic echinococcosis, which affects primarily traditional pastoralists in low socio-economic settings, has a long latency period and may form incubatory cysts in numerous organs in the body, most notably the liver and the lungs, causing hepatic cystic echinococcosis (HCE) and pulmonary CE, respectively.^2,3,4,5^

HIV-associated immune deficiency is considered a risk factor for the acquisition and progression of opportunistic infections.^6,7,8^ In countries with overlapping geographical distributions of parasitic infestations and HIV, it has been speculated that the rapid spread of HIV may be linked to increased susceptibility to HIV in patients with helminthic infestations.^7,8,9,10^ Although evidence remains limited, anecdotal conjecture and observations from case reports and small case series have led to the hypothesis that the corollary may also be true, namely that HIV-associated immunosuppression may result in a greater parasitic burden and more severe helminthic disease.^8,10,11,12,13,14^ Hepatic CE is endemic in many countries with high prevalences of HIV, including South Africa (SA).^12,13^ Comparative data on HCE treatment outcomes in HIV-positive (HIV+) and HIV-negative (HIV−) patients are limited to small case series.^11,14,15,16,17,18,19,20,21^ From the sparse data available, it has been suggested that HCE patients with HIV co-infection present with a more complicated clinical course, necessitating more frequent emergency surgery and/or pre-operative endoscopic or percutaneous interventions, potentially leading to an increased risk of post-operative complications and mortality.^4,11,14,15,16,18,19,20,22,23,24^

Imaging plays an important role in the management of HCE. Ultrasound is the imaging method of choice for screening and for confirming the diagnosis, as clinical findings are often non-specific and serology is unreliable because of low sensitivity.^3,4,25,26^ Cross-sectional imaging with contrast-enhanced CT (CE-CT) and MRI is invaluable in the pre-operative assessment of patients, to map the extent of the disease in the liver, to depict the proximity of cysts to vital structures such as major blood vessels and bile ducts, to identify complicated cysts such as those with cyst-biliary communications and to exclude extrahepatic CE.^3,26,27,28^ In clinical practice, the initial suspicion of a complicated presentation is based on clinical findings (history and physical examination) but confirmed on imaging.

Classification systems for grading the disease are based on imaging.^3,29,30^ The Gharbi classification is based on ultrasound findings, whereas the WHO classification, originally based on ultrasound, is often informally used for other imaging modalities and for comparisons of findings from multiple imaging procedures in the context of HCE.^29,30,31^ Imaging is also helpful in detecting or assessing post-interventional complications.^28^ No dedicated comparative studies have been published on imaging characteristics in HCE patients, with and without HIV co-infection. Based primarily on case reports, it has been hypothesised that CE with HIV co-infection is associated with larger cysts, more frequent multilocular disease, accelerated cyst growth and a higher prevalence of WHO type CE2 and CE3 cysts.^11,15,20,21^ Furthermore, wider cyst dissemination, with cysts more often found in unusual locations, has been reported.^4,8,11,14,15,18,19,20,21^ In case studies, biliary obstruction, secondary infection and cyst rupture were reported as more common in patients with HCE and HIV co-infection.^15,16,19,22,23,24^

South Africa, burdened with high prevalences of both HIV and CE, provided a large patient population with HIV-HCE co-infection which enables comparison of imaging characteristics in HCE patients, with and without HIV.^12,14,32^ The purpose of this study was to assess the previously unproven hypothesis that patients with HCE and HIV co-infection present with more advanced and/or more frequently complicated disease than HIV− patients through a comparative analysis of pre-operative imaging in the two groups.

## Research methods and design

A retrospective, descriptive study was conducted. Consecutive patients with HCE, who either had an existing positive HIV test or consented to a voluntary HIV test when presenting for HCE treatment, and who were managed in the Division of General Surgery at Groote Schuur Hospital (GSH) in Cape Town, SA, between 01 January 2011 and 30 September 2023, were assessed for inclusion in the study. Patients without at least one available cross-sectional abdominal imaging modality were excluded.

A standardised imaging protocol was not applied in this study because patients were managed over an extended period during which imaging availability and clinical practice evolved. Imaging investigations were performed according to clinical requirements, availability of modalities and evolving practice patterns during the study period. For patients who underwent surgery, all imaging, from the first diagnostic to the final pre-operative imaging, was assessed. For non-operated patients, all available imaging, up to the decision not to operate, were assessed. The imaging was independently reviewed by two specialist radiologists who were blinded to all clinical information, including HIV status. Differences in assessment were resolved through a third blinded specialist.

Hepatic cyst characteristics were based on the collated findings from all pre-operative imaging modalities. Extrahepatic cysts were documented. Findings were evaluated according to the Gharbi and WHO classification systems, summarised in the footnote of Table 4.^29,30^ Staging of disease according to the Gharbi classification included only patients who, in addition to cross-sectional imaging, had ultrasound examinations.^30^ For the WHO staging, findings from all performed imaging modalities were collated. In patients with multiple cysts, the cyst with the most advanced stage was used for classification, and the largest hepatic cyst was used to record size.^29,31^

Background demographic information was collected from an ethics-approved, prospectively maintained institutional registry. Differences in radiological findings and/or cyst characteristics were assessed for the total cohort and as per the HIV status. The research adhered to the Strengthening the Reporting of Observational Studies in Epidemiology (STROBE) reporting guidelines for cohort studies and used terminology consistent with the international consensus document for Echinococcoses.^33,34^

### Statistical analysis

Data were obtained using a REDCap (Research Electronic Data Capture) database hosted by the University of Cape Town, Cape Town, South Africa. REDCap is a secure, web-based data capture platform developed by Vanderbilt University, Nashville, Tennessee, USA. Statistical analyses were performed using R version 4.3.0 (R Foundation for Statistical Computing, Vienna, Austria) within RStudio (Ocean Storm release; Posit Software, PBC, Boston, Massachusetts, USA). Functions from the KODAMA R package (version 2.4, CRAN repository) were applied.^35^ Continuous variables (e.g. age and biochemical markers) were compared using the Wilcoxon rank-sum test via the ‘continuous.test’ function. Categorical variables (e.g. gender) were analysed using Fisher’s exact test via the ‘categorical.test’ function. A *p*-value less than or equal to 0.05 was considered statistically significant. Analyses were conducted using available data, with missing values excluded from the relevant comparisons. Table footnotes indicate variables for which complete data were not available for all patients.

### Ethical considerations

The CE registry and the present study received approval from the Human Research Ethics Committee (HREC) of the Faculty of Health Sciences, University of Cape Town. The ethical clearance number for the CE registry is HREC/REF: R019/2016, and the clearance number for this study is HREC REF: 660/2023.

## Results

Of the 114 HCE patients managed in the unit, 86 patients had undertaken HIV testing and met all the inclusion criteria for the study. Sixty-three (73.3%) females and 23 (26.7%) males, of whom 45 (52.3%) were HIV+, were included in the study. The prevalence of HIV was similar in females and males (54.0%; 47.8%; *p* = 0.635) (Table 1). HIV+ patients tended to present at a younger age (32 years old) than HIV− patients (38 years old) (*p* = 0.389) (Table 2).

**TABLE 1:** Demographic data for the total patient cohort and data comparison by HIV status.

Variables	Total		HIV positive		HIV negative		*P*
	*n*	%	*n*	%	*n*	%	
**Participants**	86	100.0	45	52.3	41	47.7	-
**Sex**							0.635
Female	63	73.3	34	75.6	29	70.7	
Male	23	26.7	11	24.4	12	29.3	

**TABLE 2:** Median and range for patient cohort and comparison by HIV status.

Variable	Total		HIV positive		HIV negative		*P*
	Median	Range	Median	Range	Median	Range	
Age	33	14–72	32	14–66	38	17–72	0.389
BMI†	23.6	14.8–47.3	23.4	17.5–47.3	24.7	14.8–35.2	0.361

† , Calculated in patients in whom data were reported.

Presenting symptoms were similar in the two groups, with abdominal pain being the dominant symptom in both; however, fewer HIV+ patients tended to present with a complicated HCE clinical presentation (62.2% vs. 78.0%, *p* = 0.158). Serology for CE was positive in 57.8% of patients in the HIV+ group, compared to 85.4% in the HIV− group (*p* = 0.018). Most HIV+ patients (80%) were on antiretroviral therapy (ART). Despite this, 21 patients (55.3%) had CD4+ helper T-cell (CD4+) counts < 350 cells/mm^3^, which is consistent with advanced immunosuppression, of whom 12 (31.6%), per definition, had HIV/AIDS (< 200 cells/mm^3^).

There were no differences in the imaging modalities performed in the HIV+ and HIV− patients (Table 3). The cyst characteristics in HIV+ and HIV− patients from all imaging modalities are summarised in Table 4. Single cysts were more frequent in both groups, with single unilocular cysts predominating. Cysts were present, on average, in four hepatic segments in each group. Liver segment 6 (80.0% in HIV+ vs. 48.8% in HIV−, *p* = 0.003) and segment 7 (75.6% in HIV+ vs. 53.7% in HIV−, *p* = 0.043) were more frequently involved in HIV+ patients. A lower proportion of left hemi-liver dominance was seen in HIV+ patients (14%) compared with HIV− patients (37.5%), *p* = 0.022. No other significant differences were found.

**TABLE 3:** Imaging procedures that were assessed in patients for the total patient cohort and data comparison by HIV status.

Imaging modalities	Total		HIV positive		HIV negative		*P*
	*n*	%	*n*	%	*n*	%	
Participants in the study	86	100.0	45	52.3	41	47.7	-
Chest X-ray	67	77.9	34	75.6	33	80.5	0.613
Ultrasound	32	37.2	19	42.2	13	31.7	0.375
CE-CT	83	96.5	44	97.8	39	95.1	0.603
MRI	7	8.1	1	2.2	6	14.6	0.050

**TABLE 4:** Cumulative cystic echinococcosis imaging findings data for the total patient cohort and data comparison by HIV status.

Imaging findings	Total				HIV positive				HIV negative				*P*
	*n*	%	Median	FR	*n*	%	Median	FR	*n*	%	Median	FR	
Participants in the study	86	100.0	-	-	45	52.3	-	-	41	47.7	-	-	-
Number of cysts													1.000
Single	52	60.5	-	-	27	60.0	-	-	25	61.0	-	-	-
Multiple	34	39.5	-	-	18	40.0	-	-	16	39.0	-	-	-
Hepatic cyst number combined with macroscopic cyst structure (locularity)†													0.716
Single unilocular cyst	32	37.2	-	-	17	37.8	-	-	15	36.6	-	-	-
Single multilocular cyst	19	22.1	-	-	10	22.2	-	-	9	22.0	-	-	-
Multiple unilocular cysts	17	19.8	-	-	7	15.6	-	-	10	24.4	-	-	-
Multiple multilocular cysts	18	20.9	-	-	11	24.4	-	-	7	17.1	-	-	-
Total number of cysts	-	-	1	1–30	-	-	1	1–20	-	-	1	1–30	0.623
Diameter of largest cyst	-	-	12.4	2.5–28	-	-	14.2	2.5–28	-	-	11.3	5.8–27.1	0.208
Size of the largest cyst													0.923
< 5 cm	3	3.5	-	-	2	4.4	-	-	1	2.4	-	-	-
5–10 cm	23	26.7	-	-	11	24.4	-	-	12	29.3	-	-	-
> 10 cm	60	69.8	-	-	32	71.1	-	-	28	68.3	-	-	-
Hemi-liver predominance summary													0.022
Left	21	25.3	-	-	6	14.0	-	-	15	37.5	-	-	-
Right	62	74.7	-	-	37	86.0	-	-	25	62.5	-	-	-
Affected segments	-	-	4	1–8	-	-	4	1–8	-	-	4	1–8	0.642
Seg 1	10	11.6	-	-	4	8.9	-	-	6	14.6	-	-	0.508
Seg 2	29	33.7	-	-	11	24.4	-	-	18	43.9	-	-	0.070
Seg 3	24	27.9	-	-	10	22.2	-	-	14	34.1	-	-	0.239
Seg 4	48	55.8	-	-	21	46.7	-	-	27	65.9	-	-	0.086
Seg 5	49	57.0	-	-	29	64.4	-	-	20	48.8	-	-	0.191
Seg 6	56	65.1	-	-	36	80.0	-	-	20	48.8	-	-	0.003
Seg 7	56	65.1	-	-	34	75.6	-	-	22	53.7	-	-	0.043
Seg 8	54	62.8	-	-	29	64.4	-	-	25	61.0	-	-	0.825
Patients with extrahepatic cysts	32	37.2	-	-	18	40.0	-	-	14	34.1	-	-	0.657
Extrahepatic location†													
Abdomen	24	27.9	-	-	14	31.1	-	-	10	24.4	-	-	0.631
Thorax	16	18.6	-	-	7	15.6	-	-	9	22.0	-	-	0.581
Extrahepatic cyst in both abdomen & thorax	8	9.3	-	-	3	6.7	-	-	5	12.2	-	-	0.470
Extrahepatic cyst only in abdomen	16	18.6	-	-	11	24.4	-	-	5	12.2	-	-	0.174
Extrahepatic cyst only in thorax	8	9.3	-	-	4	8.9	-	-	4	9.8	-	-	1.000
Additional findings†													
Intra-peritoneal rupture	20	23.3	-	-	9	20.0	-	-	11	26.8	-	-	0.610
Cyst infection	12	14.0	-	-	4	8.9	-	-	8	19.5	-	-	0.216
Ascites or fluid	13	15.1	-	-	9	20.0	-	-	4	9.8	-	-	0.235
Visible bile duct dilatation	12	14.0	-	-	5	11.1	-	-	7	17.1	-	-	0.538
Visible bile duct communication	10	11.6	-	-	5	11.1	-	-	5	12.2	-	-	1.000
Additional non-viable cysts	6	7.0	-	-	2	4.4	-	-	4	9.8	-	-	0.418
Signs of cyst calcification	5	5.8	-	-	1	2.2	-	-	4	9.8	-	-	0.187
Visible intraductal cysts	4	4.7	-	-	1	2.2	-	-	3	7.3	-	-	0.344
Visible obstructive mass effect on the bile ducts	4	4.7	-	-	1	2.2	-	-	3	7.3	-	-	0.344
Gharbi classification†													0.334
Type I	30	36.1	-	-	16	37.2	-	-	14	35.0	-	-	-
Type II	10	12.0	-	-	4	9.3	-	-	6	15.0	-	-	-
Type III	33	39.8	-	-	20	46.5	-	-	13	32.5	-	-	-
Type IV	10	12.0	-	-	3	7.0	-	-	7	17.5	-	-	-
WHO classification													0.521
CL	27	32.5	-	-	15	34.9	-	-	12	30.0	-	-	-
CE 1	2	2.4	-	-	1	2.3	-	-	1	2.5	-	-	-
CE 2	17	20.5	-	-	10	23.3	-	-	7	17.5	-	-	-
CE 3	26	31.3	-	-	14	32.6	-	-	12	30.0	-	-	-
CE 4	11	13.3	-	-	3	7.0	-	-	8	20.0	-	-	-

WHO, World Health Organisation; CE, cystic echinococcus; FR, full range.

Gharbi Type I, Pure fluid collection; Type II, Fluid collection with a detached membrane; Type III, Fluid collection with multiple septa and/or daughter cysts; Type IV, Hyperechoic with high internal echoes.

WHO CL, unilocular anechoic cystic lesion without any internal echoes and septations; CE 1, uniformly anechoic cyst with fine echoes settled in it, representing hydatid sand; CE 2, cyst with multiple septations giving it multivesicular appearance or rosette appearance or honey comb appearance with unilocular mother cyst: active stage of the cyst; CE 3, unilocular cyst with daughter cysts with detached laminated membranes appearing as water lily sign: transitional stage of the cyst; CE 4, mixed hypo and hyperechoic contents with absent daughter cysts, these contents give an appearance of ball of wool sign indicating the degenerative nature of the cyst.

† , Calculated in patients in whom data were reported.

**FIGURE 1:**
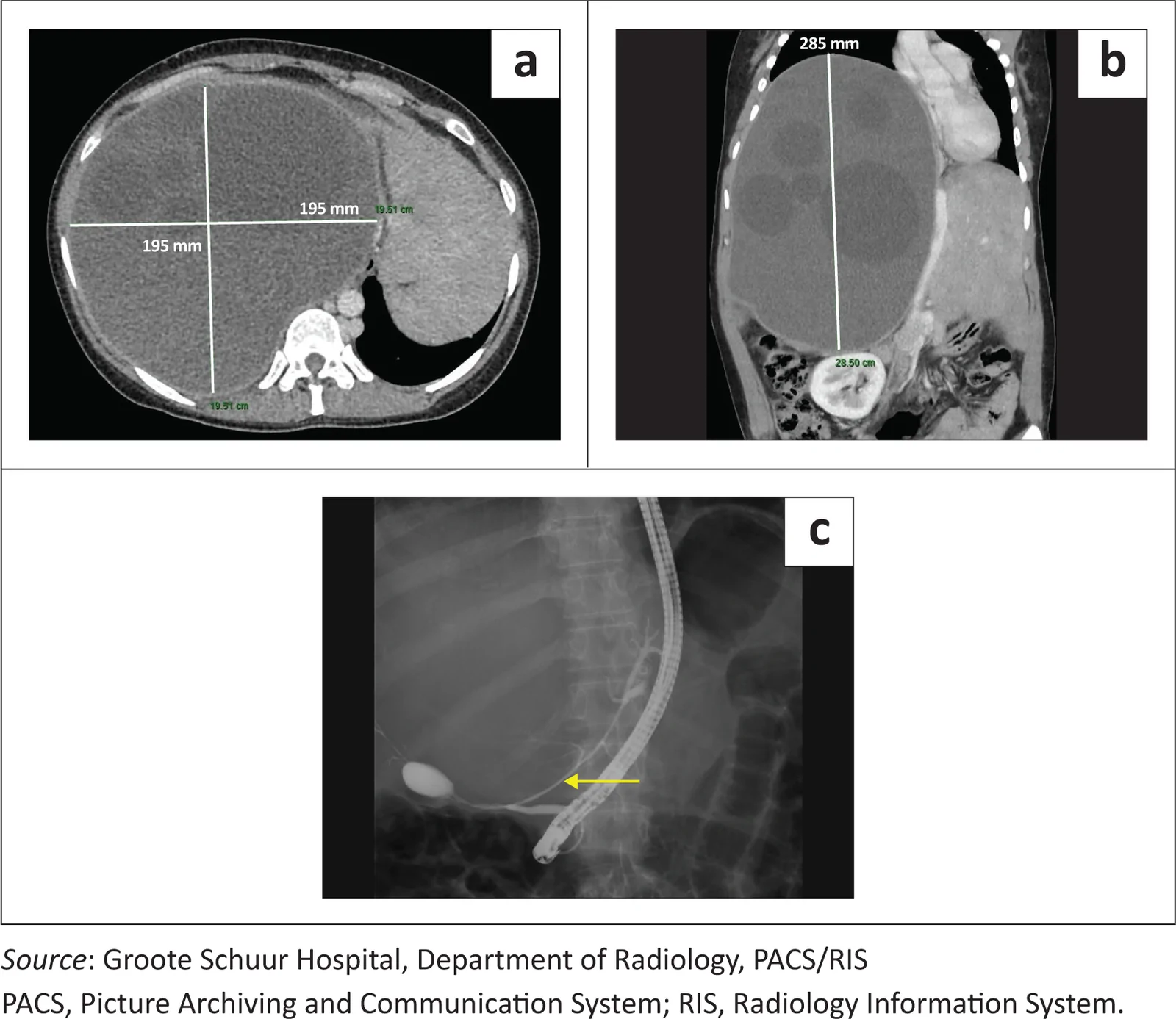
A CE-CT scan of a large cyst of the right hemi-liver measuring 195x195x285 mm (anteroposterior, transverse, craniocaudal) (a and b), in an HIV-positive patient female 29-years of age, compresses the right lung, displaces the heart into the left hemithorax and displaces the right kidney inferior and medial. An endoscopic retrograde cholangiopancreatography (c) shows the common hepatic duct compressed and stretched over the cyst (arrow).

**FIGURE 2:**
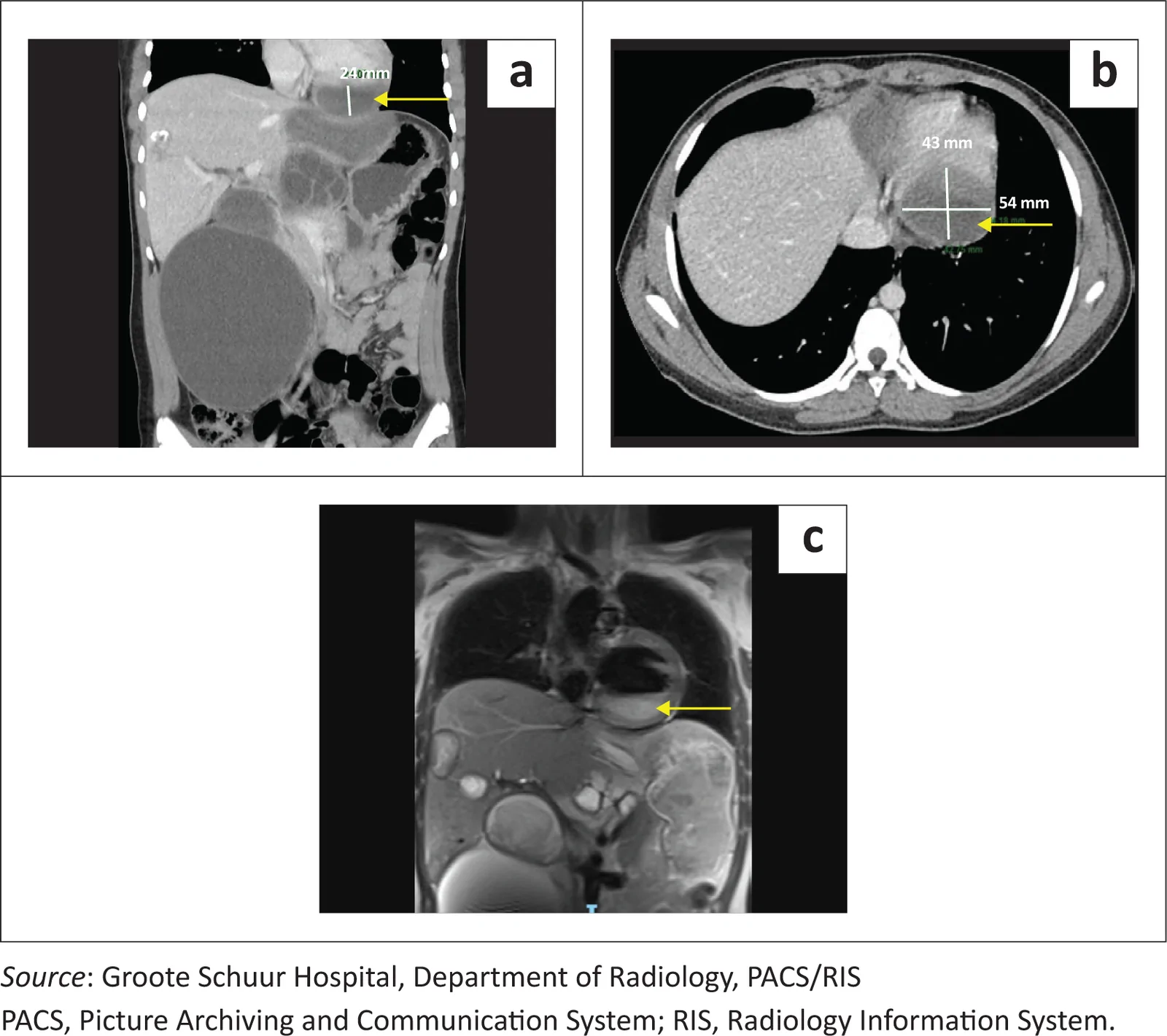
Contrast-enhanced coronal (a) and axial (b) CT and coronal T2 MRI (c) of a 17-year-old HIV-negative female patient with hepatic and extrahepatic cysts and, notably, a myocardial cyst, measuring 54 mm × 43 mm × 24 mm (anteroposterior, transverse, craniocaudal) (arrows).

The median and range of diameters of the largest hepatic cysts in each group were similar, but with a trend of larger cysts in HIV+ patients (median 14.2 cm) compared to HIV− patients (11.3 cm) (*p* = 0.208) (Figure 1). Similarities were also noted in cyst size, with the greatest diameter of the largest cyst exceeding 10 cm in both HIV+ and HIV− patients. There were statistically non-significant trends of extrahepatic cysts occurring more often in HIV+ (40.0%) than in HIV− (34.1%) patients (*p* = 0.657), and HIV+ patients having a slightly higher ratio of abdominal to thoracic extrahepatic cysts (Figure 2). Radiologically identified cyst complications were similar in terms of number and type.

**FIGURE 3:**
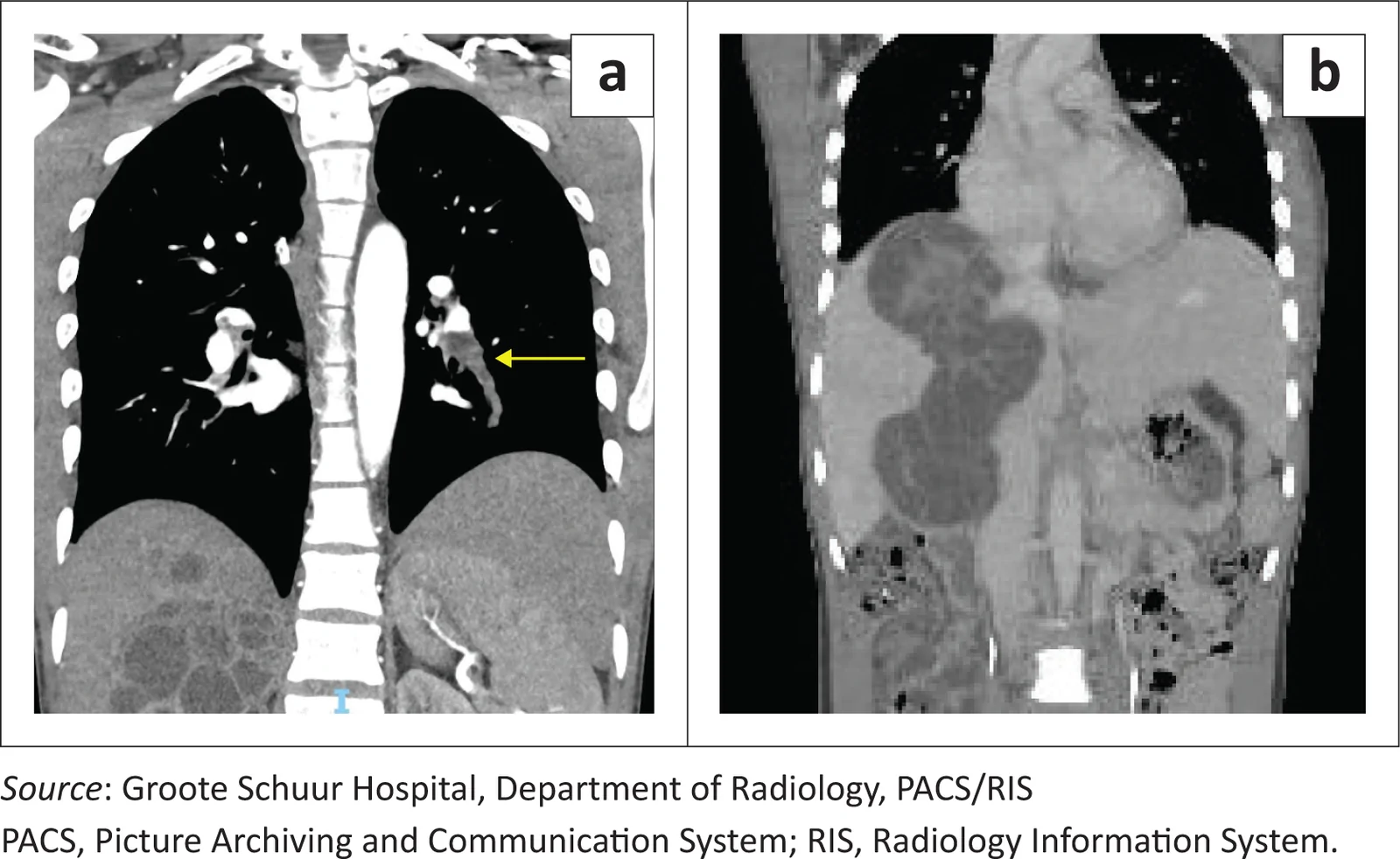
Contrast-enhanced CT images of a 24-year-old HIV-negative male who presented with a clinical picture suggesting pulmonary embolism. A contrast-enhanced CT showed multiple emboli in the right and left lower lobes (arrow) (a) and cysts in segments 4a and 8 between the inferior vena cava and the hepatic veins with suspected cyst invasion into the middle hepatic vein (b).

Gharbi Type III, followed by Type I cysts with heterogeneous echogenicity, were more common in HIV+ patients, compared to Type I followed by Type III with hypoechoic echogenicity in HIV− patients. Cystic lesion (CL) cysts followed by CE3 cysts as per the WHO classification were the most common in both groups. However, none of these differences was statistically significant (Table 4). Splenic cysts were seen in five patients in the HIV+ group but none in the HIV− group, *p* = 0.053. Other rare cyst locations were evenly spread between the groups. Extrahepatic cysts were seen in individual HIV+ patients in the pelvic fat, the duodenum, the right kidney and in Morrison’s pouch. In individual HIV− patients, cysts were seen in the inferior vena cava (IVC) and pulmonary arteries, the myocardium, the abdominal wall, the pancreas and the pelvis (Figure 3).

## Discussion

The current study assessed the hypothesis that HCE patients co-infected with HIV present with more advanced or complicated HCE disease than HIV− patients, by comparing pre-operative cyst characteristics, including size, number and macroscopic structure (locularity) between the groups. Complications detected on imaging, such as rupture, infection and biliary obstruction, as well as the presence of extrahepatic cysts, were compared in the two cohorts. Despite 21 (55.3%) of the HIV+ patients presenting with advanced HIV disease or AIDS, the results indicated no statistically significant differences in imaging findings, except for a right hemi-liver predominance in HIV+ patients (*p* = 0.022). These findings raise important questions regarding the interaction between HIV and CE and challenge previous assumptions that HIV exacerbates the severity or complexity of this parasitic disease.

The prevalence of HIV co-infection of 52.3% in the cohort was remarkably higher than the national HIV prevalence of approximately 12.7% reported in the general South African population.^36^ There was a well-documented female predominance in the total patient cohort (73.3%) with no difference between HIV+ and HIV− patients (75.6% vs. 70.7%).^13,32^ There was a non-significant trend in HIV+ patients presenting at a younger median age of 32 years compared to 38 years in HIV− patients, which may reflect faster cyst growth or earlier acquisition of HCE in HIV+ patients. This is supported by a trend, although not significant, towards a larger median cyst diameter of 14.2 cm in HIV+ patients, compared to 11.3 cm in HIV− patients. Single unilocular cysts, measuring over 10 cm in diameter, dominated the total cohort and were similar in both groups. This is consistent with the general presentation of HCE, which is often characterised by large, slow-growing cysts that can remain asymptomatic for years.^3,26,37^ Multiple cysts have been reported in 20% – 40% of CE patients, which is consistent with our findings.^26^ Typically, 80% of cysts in HCE patients arise in the right hemi-liver, possibly because of the larger parenchymal volume and higher portal venous volume supply, compared to the left hemi-liver.^3,27^ An interesting finding in the current study was that this trend was present in HIV+ patients, but less so in HIV− patients. HIV+ patients had a significantly higher prevalence of cysts in liver segments 6 and 7 (*p* = 0.003 and 0.043), which was also reflected in the right hemi-liver dominance mentioned earlier. This could indicate a subtle difference in the anatomical spread of cysts in immunocompromised individuals, though the clinical significance of this remains unclear.

While these differences in anatomical distribution may be statistically significant, they did not correlate with more severe disease or more complications detected on imaging. In the literature, HCE complications on presentation range between 30% and 60%.^27,37,38^ No significant differences in complications were found between the two groups. HIV+ and HIV− patients had similar rates of cyst infection, rupture and bile duct obstruction, which does not support findings in previous reports.^15,16,19,20,22,23^ It is possible that ART may have modified the effects of HIV on the progression of HCE.^6,10^ Unfortunately, the number of patients was insufficient to allow a sub-analysis comparing findings in ART-compliant versus non-treated patients.

Multi-organ involvement has also been reported in 20% – 40% of CE patients.^26^ The present study found that extrahepatic cysts occurred in 37.2% with similar proportions in each group, but with some differences in the location of the cysts. HIV+ patients were more likely to have abdominal extrahepatic cysts, while HIV− patients had a slightly higher prevalence of intra-thoracic cysts. According to published research, splenic cysts are documented to occur in fewer than 2% of CE patients.^39^ Notably, splenic cysts were detected in five (11.1%) of the HIV+ patients but none in the HIV− group, which may indicate a unique pattern of disease dissemination in immunocompromised individuals, and may warrant further investigation in larger cohorts. There were no statistical differences in the cyst types as categorised according to either the Gharbi or the WHO classifications.

The study underscores the central role of imaging in the management of HCE, especially in the context of HIV co-infection. Firstly, the diagnostic role of imaging is particularly relevant in HIV+ patients. Serology for echinococcosis was positive in only 57.8% of HIV+ patients, compared to 85.4% of HIV− patients (*p* = 0.018), supporting previous reports that serological testing is less reliable in HIV+ patients, potentially as a result of a compromised immune response in activating antibodies.^8,13,18^ Secondly, clinicians should be aware that HIV+ patients in this cohort tended to present at a younger age although the reasons for this observation remain unclear. There was also the potential need for more extensive imaging to detect extrahepatic disease in unusual locations. Thirdly, while the importance of testing for opportunistic infections in HIV+ patients is well known, it is increasingly clear that HIV testing should take place when a suspected CE cyst is seen on imaging in populations with a high prevalence of HIV. To the authors’ knowledge, this is the first cohort study of HCE and HIV co-infection with detailed imaging findings, confirming or refuting prior observations from case reports and small case series.

### Study limitations

The single-centre, retrospective study design reduces the generalisability of the findings. The referral of predominantly symptomatic and/or complicated HCE patients to our tertiary centre may have introduced selection bias and limited the generalisability of the findings to asymptomatic patients. The chronic and often asymptomatic initial nature of both HCE and HIV precluded the determination of the temporal relationship between the two infections. HIV testing was voluntary under South African legislation, and not all patients underwent screening, which may have introduced additional bias.^40^ The retrospective study design, absence of serial imaging and lack of a standardised imaging protocol limited assessment of cyst growth over time. Furthermore, many patients required prompt therapeutic intervention following diagnosis, reducing opportunities for longitudinal imaging assessment. As a tertiary referral centre, some imaging studies were performed at referring primary or secondary healthcare facilities, resulting in variability in imaging availability and completeness. In addition, our resource-limited healthcare environment restricted access to MRI, which was reserved for selected patients. Finally, incomplete data, including duration of ART and treatment compliance, may have influenced the findings.

## Conclusion

Despite the high prevalence of HIV co-infection in this cohort, HCE patients with HIV did not demonstrate more advanced and/or complicated disease on imaging than HIV− patients. These findings challenge previous assumptions regarding the effect of HIV-associated immunosuppression on the radiological manifestations of HCE. The disproportionately high HIV prevalence within the cohort nevertheless warrants further investigation into possible interactions between the two diseases. Although not statistically significant, trends towards a younger age at presentation and larger cyst size in HIV+ patients may merit further exploration in larger studies. Imaging remains the cornerstone of HCE diagnosis and management, particularly in HIV+ individuals, where serological testing may be less reliable. Larger multicentre prospective studies are needed to confirm these findings and to explore the long-term impact of HIV co-infection on the presentation and progression of HCE.
